# Functional Loss of *Bmsei* Causes Thermosensitive Epilepsy in Contractile Mutant Silkworm, *Bombyx mori*

**DOI:** 10.1038/srep12308

**Published:** 2015-07-22

**Authors:** Hongyi Nie, Tingcai Cheng, Xiaofeng Huang, Mengting Zhou, Yinxia Zhang, Fangyin Dai, Kazuei Mita, Qingyou Xia, Chun Liu

**Affiliations:** 1 State Key Laboratory of Silkworm Genome Biology, Chongqing 400716, China; 2 the Key Sericultural Laboratory of the Ministry of Agriculture, Southwest University, Chongqing 400716, China; 3 College of Bio-Technology, Southwest University, Chongqing 400716, China; 4 College of Bee Science, Fujian Agriculture and Forestry University, Fujian Fuzhou 350002, China

## Abstract

The thermoprotective mechanisms of insects remain largely unknown. We reported the *Bombyx mori* contractile (*cot*) behavioral mutant with thermo-sensitive seizures phenotype. At elevated temperatures, the *cot* mutant exhibit seizures associated with strong contractions, rolling, vomiting, and a temporary lack of movement. We narrowed a region containing *cot* to ~268 kb by positional cloning and identified the mutant gene as *Bmsei* which encoded a potassium channel protein. Bmsei was present in both the cell membrane and cytoplasm in wild-type ganglia but faint in *cot*. Furthermore, Bmsei was markedly decreased upon high temperature treatment in *cot* mutant. With the RNAi method and injecting potassium channel blockers, the wild type silkworm was induced the *cot* phenotype. These results demonstrated that *Bmsei* was responsible for the *cot* mutant phenotype and played an important role in thermoprotection in silkworm. Meanwhile, comparative proteomic approach was used to investigate the proteomic differences. The results showed that the protein of Hsp-1 and Tn1 were significantly decreased and increased on protein level in *cot* mutant after thermo-stimulus, respectively. Our data provide insights into the mechanism of thermoprotection in insect. As *cot* phenotype closely resembles human epilepsy, *cot* might be a potential model for the mechanism of epilepsy in future.

The ability to sense and respond to the ambient temperature is crucial for the survival and fitness of all animals. Temperature influences the cellular metabolism and muscular contractions, and modulates many aspects of neuronal function from conduction velocity to the refractory period^1^,^2^,^3^. Insects make up a substantial proportion in the animal kingdom. Most insects face adverse conditions in which the temperature is outside their physiological limits, so insects have evolved a phenomenal ability to adjust their temperatures using different strategies^4^. The thermoprotection of insects is an important physiological characteristic that constitutes part of their adaptability to the environment. Heat shock proteins and sorbitol are reported to play roles in the thermoprotective mechanisms of some insect species^5^,^6^,^7^,^8^. Although thermoprotection has been reported in many insects, including locusts, mealworms, beetles, moths, ants, flies, and wasps^9^,^10^,^11^, our understanding of the mechanisms that protect insects from the effects of high temperatures is limited.

The study of mutants has played a vital role in understanding the mechanisms underlying physiological and behavioral changes^12^,^13^,^14^,^15^. A *Drosophila* mutant displaying unique behaviors after mechanical stimulation (“bang”), including intense abnormal contractions, wing flapping, proboscis extension, and leg shaking, has become a potentially powerful system for modeling human pathologies^16^. The *dao* mutant, which manifests bouts of uncontrolled motor activity during flight, followed by paralysis, when it is suddenly shifted to 37 °C, has shown that the Dao protein is crucial for establishing the proper level of neuronal membrane excitability^17^. More than 400 silkworm mutants are preserved in the silkworm gene banks of China and Japan. About 40 mutant genes have been identified so far by positional cloning or positional cloning combined with other methods. The genes identified are responsible for larval body color (*sch*)^18^, the larval stripe (*cts*)^19^, blood color (*Y* and *rb*)^20^,^21^, cocoon color (*Gb*)^22^, pupal and moth color^23^,^24^ (*so* and *mln*), pathology mutants (*nsd-1* and *nsd*-*2*)^25^,^26^, and moultinism (*mod* and *nm-g*)^27^,^28^. These mutants are valuable resources for studying the genetics and gene functions of the silkworm *Bombyx mori*. The genes responsible for these mutants have been identified and the mutant mechanisms determined, promoting the development of the silkworm as a Lepidoptera model organism.

The mutant genes cloned to date are involved in the morphology, physiology, and pathology of the silkworm, but not in its behavior. In this study, a rare behavioral mutant, contractile (*cot*), was investigated. The recessive mutant *cot* is controlled by a single gene located at locus 25.0 centimorgan (cM) on silkworm genetic linkage group 15^29^. In our previous study, we showed that *cot* is a thermo-sensitive paralysis mutant, which manifests when it is exposed to 35 °C, at which temperature it displays strong contractions, rolling, vomiting, and a temporary lack of movement after manual stimulation, followed by slow recovery^30^. However, the corresponding mutant gene was not isolated. Therefore, *cot* is an excellent mutant with which to study relationship between behaviors and the molecular mechanism that protect insects from the effects of high temperatures. Recently, it has been reported that the silkworm is a potential animal model for human diseases^31^. As *cot* phenotype resembles human epilepsy, it might be a valuable resource for the further investigation of the mechanism underlying epilepsy.

We used positional cloning in the *cot* mutant to identify the *B. mori seizure* gene (*Bmsei*), which contains a 15-bp deletion in the fifth intron, generating three abnormal splicing isoforms around the fifth exon in the *cot* mutant, which produce nonfunctional proteins. Using combinations of immunofluorescence, immunoblotting, RNA interference (RNAi) and injection of a potassium channel blocker, these results demonstrate that *Bmsei* was responsible for the *cot* mutant phenotype. A comparative proteomic analysis was used to identify the proteins differentially expressed in the *cot* mutant when exposed to high temperature. Our results suggest that the *cot* mutant is a good model in which to study the behavior and the molecular mechanism of thermoprotection in insects.

## Results

### Novel Method Identifies *cot* Mutants with Different Genotypes

**Figure 1:**
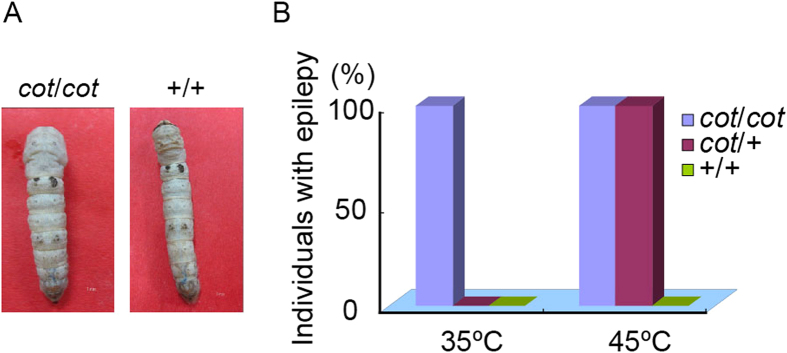
Phenotype of the *cot* mutant. (**A**) Artificial stimulation with high temperature induced the *cot* seizure phenotype. (**B**) Behavioral response to high temperature in the *cot*/*cot*, *cot*/+, and +/+ strains. *cot/cot* larvae display the epilepsy phenotype at 35 °C; *cot/+* larvae displayed the phenotype at 45 °C. WT (+/+) larvae without the epilepsy phenotype. Ninety individuals were investigated per genotype group.

It was necessary to separate the *cot* mutants with different genotypes before positional cloning. The *cot* mutant was first induced by rubbing the silkworm larvae by hand^29^. However, because the periods and strength of rubbing differ, it was difficult to discriminate the *cot/cot* and *cot/+* genotypes in backcrossed larvae (BC_1_) with this method. Recently, we found that *cot* is a temperature-sensitive mutant^30^. To determine the appropriate temperature at which to distinguish the *cot/cot* and *cot/+* genotypes, larvae of different genotypes (*cot/cot*, *cot*/+ and +/+) were subjected to different temperatures, ranging from 25 °C to 48 °C. All the *cot/cot* larvae showed the epilepsy phenotype when the temperature was increased to 35 °C, whereas the *cot/+* genotype showed a slightly epilepsy phenotype at 45 °C (Fig. 1). However, the WT (+/+) did not show the epilepsy phenotype, even at 48 °C. These results suggest that *cot/cot* is incompletely dominant because the *cot*/+ heterozygote showed the *cot/cot* phenotype at 45 °C. This offers an accurate method for distinguishing the *cot/cot* genotype from the *cot/+* genotype in BC_1_ groups.

### Positional Cloning of *cot*

**Figure 2:**
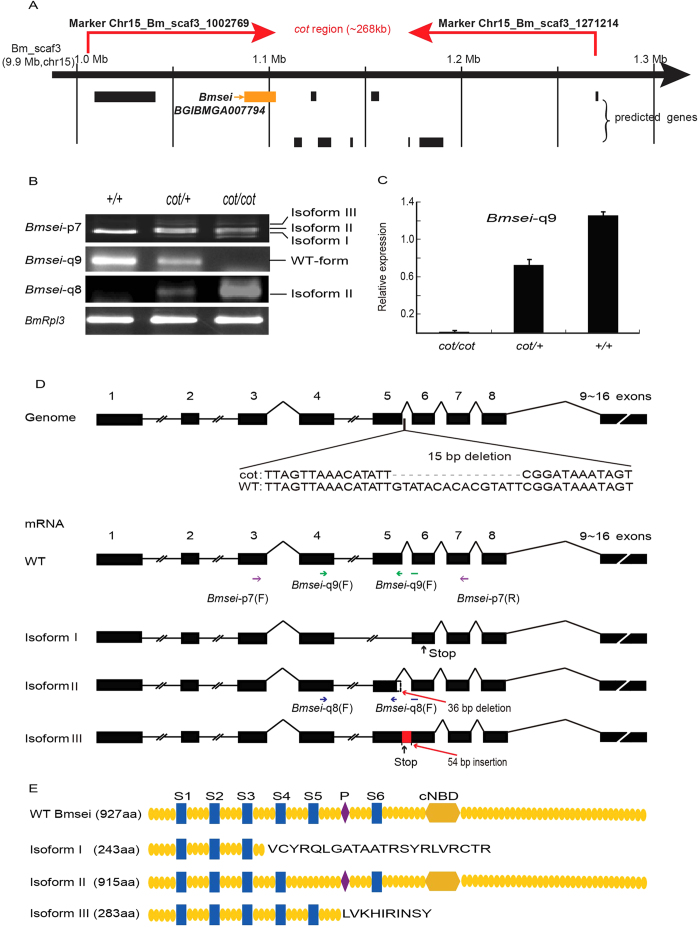
Positional cloning of the *cot* locus. (**A**) Physical map showing the outcome of the linkage analysis using 1211 BC_1_ individuals. The *cot* locus was narrowed to the genomic region flanked by the SNP markers chr15_Bm_scaf3_1271214 and chr15_Bm_scaf3_1002769, as indicated by the red arrows. Putative genes predicted with SilkDB are shown below the map, and *Bmsei* (BGIBMGA007794) is shown with the orange arrow. (**B**) Three isoforms were detected in the head of each strain with genotype *cot/cot, cot/+* and +/+ on day 5 of fifth instar with RT–PCR using *Bmsei* primers. *Bmsei*-p7 was based on the predicted sequence of *Bmsei*. *Bmsei*-q9, which was not detected in *cot*, was based on the mRNA sequence of WT. *Bmsei*-q8, which was not detected in WT, was based on the sequence of isoform II. (**C**) Relative expression of normal mRNA of *Bmsei* was verified with qPCR in the head of three genotypes (*cot*/*cot*, *cot*/+ and +/+) on day 5 of fifth instar. (**D**) The 15-bp *Bmsei* deletion in the fifth intron splicing region and the abnormal *Bmsei* transcripts in the *cot* mutant were detected by sequencing the genome and coding sequence (CDS). Isoform I lacks the entire fifth exon and causes a premature stop codon at nucleotide 733 of the CDS and encodes an aberrant protein lacking the fourth, fifth, and sixth transmembrane domains, the pore (P) region, and the cyclic nucleotide-binding domain (cNBD). Isoform II has a 36-bp deletion at the 3′ end of the fifth exon, creating a 12-amino-acid deletion in the fifth transmembrane domain of the protein. Isoform III is not spliced in the fifth intron, resulting in a 54-bp insertion in the fifth intron at the 3′ end of the fifth exon, producing a stop codon at nucleotide 853 of the CDS. Purple arrow, green arrow and blue arrow indicate the position of three primers (*Bmsei*-p7, *Bmsei*-q9 and *Bmsei*-q8), respectively. Black arrows in isoforms I and III denote premature stop codons. (**E**) Predicted structures and domains of *Bmsei* protein and isoforms.

To identify the gene responsible for the *cot* mutant, we performed positional cloning using BC_1_ individuals from crosses between a *cot*/*cot* ♀ and an F_1_ ♂ (*cot/cot* × +/+). Using 1,211 BC_1_ individuals, we mapped the *cot* locus to within a ~268-kb region on the scaffold Bm_scaf3 (chromosome 15) between single-nucleotide polymorphism (SNP) markers chr15_Bm_scaf3_1271214 and chr15_Bm_scaf3_1002769. Ten genes in the silkworm genome database were predicted to occur within this region (Fig. 2A; Supplementary information, Table S1). Of these, we focused on *BGIBMGA007794*, a gene that encodes an ion channel. BGIBMGA007794 shows a high degree of homology with *Drosophila* seizure (sei), which encodes an ether-à-go-go-related (Erg)-type potassium channel in humans (hERG) (Supplementary information, Fig. S1). We named this gene *Bmsei*. Bmsei has six transmembrane domains, a pore (P) region, and a cyclic nucleotide-binding domain (cNBD), predicted with the SMART and SOSUI programs^32^. In *Drosophila*, mutations in *sei* cause a temperature-sensitive paralytic phenotype^33^. The *cot* phenotype is similar to the *Drosophila sei* mutant, suggesting that *Bmsei* is a candidate for the mutant *cot* gene. In the WT strain, *Bmsei* contains 16 exons, 15 introns, and a 2784-bp open reading frame. Compared with the WT strain, at least three alternative splicing isoforms were identified in the cDNAs from the heads of *cot/cot* mutant on the day 5 of fifth instar, using RT–PCR and sequence analyses (Fig. 2B,D,E). Isoform I completely lacks the fifth exon and causes a premature stop codon at nucleotide 733, thus encoding an truncated protein lacking the fourth, fifth, and sixth transmembrane domains, the P region, and the cNBD domain. Isoform II has a 36-bp deletion at the 3′ end of the fifth exon, resulting in a deletion of 12 amino acids in the fifth transmembrane domain. Isoform III contains the fifth intron and a premature stop codon at nucleotide 853, but lacks the P region, the sixth transmembrane domain, and the cNBD domain. Data are deposited at the National Center for Biotechnology Information GenBank and the GenBank accessible number for WT and the mutant sequences (Isoform I, Isoform II and Isoform III) of Bmsei are KR081241, KR081242, KR081243 and KR081244, respectively.

All isoforms are caused by abnormal splicing around the fifth exon. Genomic sequencing of *Bmsei* in the *cot* mutant showed a 15-bp deletion in the fifth intron (Fig. 2D). This deletion is unique to the *cot* mutant and was not found in 11 other silkworm strains (Supplementary information, Fig. 2). RT–PCR was used to determine the expression of the WT and isoform II mRNAs, and showed that normal transcripts were highest in the WT (+/+), were not detected in the *cot/cot* genotype, and were expressed at an intermediate level in the *cot/+* genotype. These levels of normal *Bmsei* expression correlate inversely with the level of abnormal behavior (Fig. 2B). These results were confirmed in the head of *cot/cot*, *cot*/+ and +/+ strain with quantitative RT–PCR (qRT–PCR) on the day 5 of fifth instar (Fig. 2C). Isoform II expression was highest in the *cot/cot* genotype, with no expression in WT (+/+) and intermediate expression in the *cot/+* genotype. These results also suggest that silkworms with the *cot/cot* genotype produce no normal *Bmsei* mRNA.

### Bmsei Localizes in the Cell Membrane and Cytoplasm of Ganglia

To examine the expression of *Bmsei*, RT–PCR was performed on different tissues of day 5 of fifth instar larvae and in different developmental stages of the *cot/cot* mutant and WT strain. *Bmsei* is highly expressed in the heads, anterior and middle silk glands, and ganglia (Supplementary information, Fig. S3). It is expressed from the late embryo stage and through the larval stages (Supplementary information, Fig. S3). The spliced isoforms were also detected in the *cot/cot* mutant.

**Figure 3:**
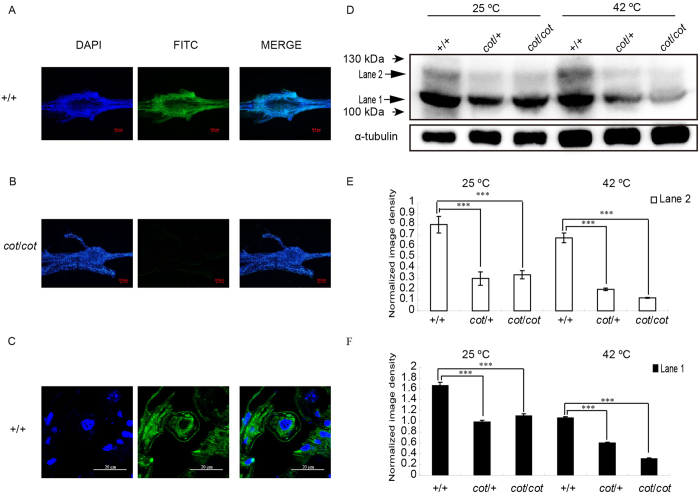
Expression and localization of Bmsei in the ganglia with immunofluorescence and immunoblotting. Immunofluorescent signal for Bmsei in whole ganglia of +/+ larvae on day 5 of fifth instar (**A**), with a weak signal in *cot/cot* larvae (**B**). (**C**) Immunofluorescence in paraffin sections of *+/+* ganglia on day 5 of fifth instar. Bmsei was localized in the cell membranes and cytoplasm. Green, positive signal; blue, DAPI staining of the nuclei. (**D**) Immunoblotting of Bmsei in the total proteins from new larvae of the three genotypes under normal conditions (25 °C) and after temperature stimulation (42 °C, 5 min). Bmsei appeared as two bands (lanes 1 and 2) in the WT. Both bands were significantly less intense (lower abundance) in the samples from *cot/cot* and *cot/+* individuals than in samples from the WT (E and F). ***P < 0.001. Error bars depict s.e.m.

An immunofluorescence analysis of whole ganglia showed that Bmsei is strongly expressed in WT (Fig. 3A), but weakly expressed in the *cot* mutant (Fig. 3B). To further determine the location of Bmsei, immunofluorescence analysis of ganglia sections from day 5 of fifth instar WT larvae showed Bmsei in the cell membrane and cytoplasm (Fig. 3C). Immunoblotting showed two Bmsei bands of around 100 kDa (lane 1 and lane 2) in samples of +/+ (WT ), *cot*/+, and *cot*/*cot* larvae maintained at room temperature (25 °C) or treated with high temperatures (42 °C, 5 min), but the expression of the two bands was markedly reduced in the *cot*/+ and *cot*/*cot* genotypes (Fig. 3D). The expression of the two bands was reduced in all three strains treated at 42 °C, but was significantly more strongly reduced in the *cot*/*cot* mutant compared with its expression in the WT (+/+) (Fig. 3D–F), suggesting that high temperature markedly accelerates the degradation of the Bmsei protein in the *cot* mutant.

### Knockdown of *Bmsei* induces the *cot* phenotype

**Figure 4:**
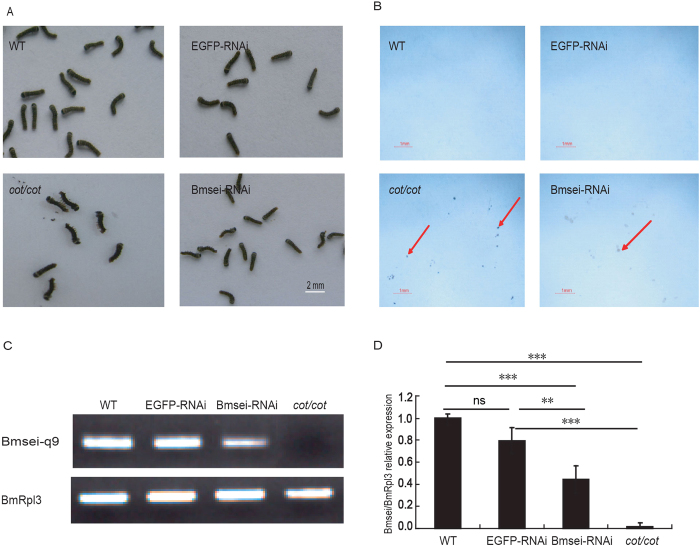
Knockdown of *Bmsei* with RNAi in WT. (**A**) RNAi directed against *Bmsei* induced the *cot* phenotype in WT larvae at 42 °C. The larvae showed contraction and rolling. (**B**) Larvae were injected with *Bmsei* dsRNA, and the *cot/cot* mutant vomited midgut juice on white paper at 42 °C. WT (no injection) and WT injected with *EGFP* dsRNA did not display this phenotype. Red arrows show the vomited midgut juices. (**C**) Expression of *Bmsei* detected with RT–PCR (*Bmsei*-q9). (**D**) Expression of *Bmsei* normalized to *Rpl3* in the qRT–PCR analysis (*Bmsei*-q9). **P < 0.01, ***P < 0.001. ns, not significant. Error bars depict s.e.m. (n = 3).

There are both deletion and insertion mutations of *Bmsei* among the *cot* mutant, which produce truncated proteins and incomplete proteins. *Bmsei* expression was knocked down with RNAi to determine whether the partial loss of Bmsei function generates the *cot* phenotype. Based on the expression profile of *Bmsei* during embryogenesis (Supplementary information, Fig. S3), *Bmsei* double-stranded RNA (dsRNA) was synthesized and injected into WT eggs within 8 h of oviposition. After hatching, the movement of the neonatal larvae injected with *Bmsei* dsRNA was similar to that of the control group injected with enhanced green fluorescent protein (*EGFP*) dsRNA at room temperature (Supplementary information, Video S1). However, when exposed to 42 °C, the neonatal larvae displayed the *cot* phenotype, with shaking bodies, inability to crawl, contractions creating S- or L-shaped bodies, falling down (Fig. 4A), and vomiting gut juice (Fig. 4B). When the temperature was reduced to room temperature, the larvae slowly recovered. The WT and *EGFP*-dsRNA-injected larvae (control groups) treated with high temperature did not display abnormal phenotypes. qRT–PCR showed that *Bmsei* expression was markedly reduced after dsRNA injection to only half of the level in the control (Fig. 4C,D). This result indicates that the knockdown of *Bmsei* expression induces the *cot* phenotype.

### Inducing and Partly Rescuing the *cot* Phenotype

To determine whether blocking the potassium channels induces the *cot* phenotype, two potassium channel blockers, tetraethylammonium chloride (TEA) and BaCl_2_, were administered to WT larvae and moths (Supplementary information, Fig. S4). The fifth instar larvae given 500 mM TEA vomited gut juice and shook vigorously and this phenotype increased at higher TEA concentrations (Fig. S4A). The wings of the moths were slightly erect after treatment with 200 mM TEA and completely erect when treated with 1000 mM TEA (Fig. S4B). The abnormal behavior produced by injecting the larvae and moths with TEA was similar to the behavior observed in *cot* larvae exposed to high temperature (42 °C). As reported earlier, BaCl_2_ blocks the inward rectifying potassium current^34^. After BaCl_2_ was administered to larvae and moths, the larvae vomited gut juice at 500 mM BaCl_2_ and displayed a more marked body shaking phenotype at 1000 mM BaCl_2_ (Fig. S4C). The moths’ legs contracted slightly at 200 mM BaCl_2_ and closed completely at 1 M BaCl_2_ (Fig. S4D), which is similar to the phenotype of *cot* moths exposed to high temperature (42 °C). These results indicate that blocking the inward rectifying potassium channels induces the *cot* phenotype, even at room temperature.

Although the mechanism is not well understood, aminoglycoside antibiotics, such as gentamicin, restore the functional expression of truncated hERG channels^35^. We rescued the *cot* mutant phenotype by injecting different concentrations of gentamicin into *cot* larvae and moths. After the gentamicin treatment, the larvae could withstand the high-temperature stimulus longer than the controls injected with ddH_2_O or with no injection; the wings of the *cot* mutants were nearly horizontal and resembled the WT as the dose of gentamicin was increased at high temperature (42 °C) in the silkworm moths (Supplementary information, Fig. S5). This result suggests that gentamicin partly rescues the *cot* phenotype.

### Proteomic Analysis of Proteins Differentially Expressed in the *cot* Mutant

**Figure 5:**
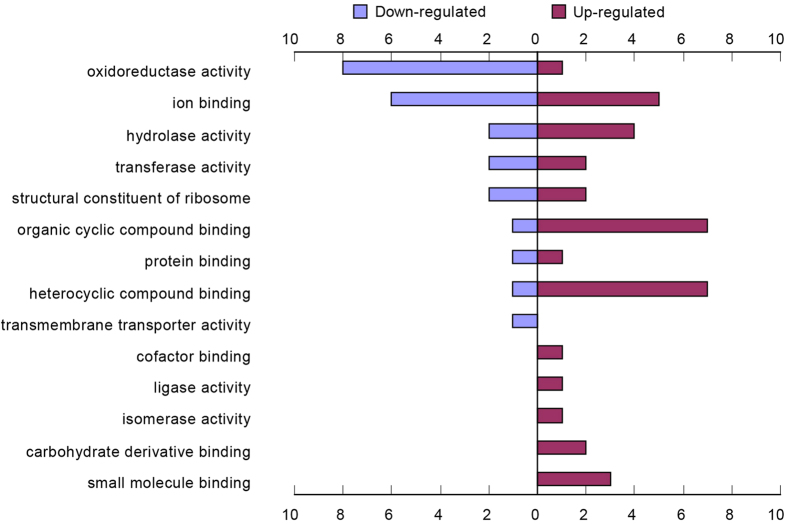
Functional categories of proteins differentially expressed in the *cot* mutant when exposed to high temperature (42 °C). The differentially expressed proteins are categorized by molecular function. Bars, numbers of genes.

The results described above indicate that *Bmsei* is the gene responsible for the *cot* mutation in the silkworm, *B. mori*. To obtain a global view of proteome changes caused by the mutation of the Bmsei protein, a comparative proteomic analysis was used to investigate the differences in protein level in neonatal larvae of a *cot* mutant strain after high-temperature treatment (42 °C, 5 min) compared with that in two control strains (*ok* strain and WT strain) using liquid chromatography–tandem mass spectrometry (LC–MS–MS). Based on the expression intensity ratios between the high-temperature-treated group and the control group in the three strains (*cot*/*cot*, *cot*/+, and +/+), 61 proteins with prominent changes were identified in the *cot* mutant after high temperature treatment, including 23 up-regulated and 38 down-regulated proteins (Supplementary information, Table S2). Among them, the protein of Hsp-1 decreased most significantly in down-regulated proteins and the most obviously up-regulated protein was Troponin I (TnI). We then analyzed whether the proteins differentially expressed in the *cot* mutants were enriched in specific molecular functions, using Blast2GO (Fig. 5; Supplementary information, Table S3). Most proteins down-regulated in the *cot/cot* strain were involved in oxidoreductase activity and ion binding. Most proteins up-regulated in the *cot/cot* strain were involved in organic cyclic compound binding, heterocyclic compound binding and ions. The proteins specifically up-regulated in the *cot/cot* strain were involved in the carbohydrate derivative binding, isomerase activity, ligase activity, and cofactor binding.

## Discussion

In this study, we identified and characterized the gene at the *cot* locus that causes a rare behavioral mutant in *B. mori*. The *cot* mutant can be identified by touching the larvae^29^. However, the identification of the *cot* mutant has been ambiguous in previous studies because the level of the *cot* phenotype induced depends on the period and strength of rubbing. Because *cot* is a temperature-sensitive epilepsy mutant, we developed a high-temperature method to stimulate phenotypes that distinguish the *cot/cot*, *cot/+*, and *+/+* genotypes. Our results may explain why the touching method of stimulating the mutant phenotype produces ambiguous results. Touch with the hand includes not only mechanical stimulation, but also thermal stimulation. The mechanical stimulus does not induce the *cot* phenotype, whereas the thermal stimulus produces the *cot* phenotype in the *cot*/*cot* and *cot*/+ genotypes. Because the human body temperature is about 37 °C, touching affected the *cot*/*cot* mutant, but not the *cot*/+ mutant.

We observed that individual WT larvae can escape from high temperatures by creeping away, whereas the temperature-sensitive *cot* mutant quickly loses its ability to crawl when treated with high temperature. In insects, the release of excess heat by different strategies allows them to avoid hyperthermia. For instance, tiger beetles change their body postures to maintain a body temperature of about 34–35 °C^36^. They can also resist high-temperature stimulation of the nervous system using surface receptors, causing physiological and behavioral changes. However, the molecular mechanisms of thermoprotection are still largely unknown. The heat shock proteins (Hsps) are crucial in the response to high temperature^5^,^7^. In our previous study, most Hsps were significantly upregulated in both the *cot* mutant strains and control strains (Dazao and *ok*) when exposed to hyperthermia for 5 min^30^. In the silkworm, Hsp-1 is up-regulated in the fat body after treatment with constant high temperature^37^. Surprisingly, Hsp-1 expression was insignificantly down-regulated in the *cot* mutant after high-temperature treatment. The *cot* mutants did not show the seizure phenotype at room temperature, even when they contained the mutated *Bmsei* protein. The phenotype was only induced by high temperature. Immunoblotting showed that the Bmsei protein was rapidly degraded during the high-temperature treatment. In humans, the human ether-à-go-go-related protein (hERG) interacts with HSP90^38^. High temperatures can induce oxidative stress^39^, which reflects an imbalance between pro-oxidants and antioxidants, leading to cell damage and tissue injury^40^. In the present study, eight proteins involved in oxidoreductase activity were down-regulated in the *cot* mutant after high-temperature treatment, indicating that these proteins play important roles in the resistance of *B. mori* to oxidative stress.

We postulated that the Hsp-1 interacts with Bmsei, facilitating its maturation and trafficking and fold. However, the temperature-sensitive paralysis of the *cot* mutant may arise from a change in the structure of the Bmsei protein or its misfolding at high temperatures; Hsp-1 cannot interact with the mutated Bmsei, producing an abnormal K^+^ current in cells, and further affects the transmission of signals to nerves. The abnormal transmission of signals might affect expression of genes involved in oxidoreductase activity, causing the cell’s resistance to oxidative stress was reduced, and induce the seizure phenotype in the organism at high temperature.

Because Bmsei is abnormally spliced around the fifth exon, producing nonfunctional proteins, the mutated Bmsei protein is markedly reduced by accelerated protein degradation using Immunoblotting in the *cot* mutant. We found that the expression of BGIBMGA007332 and BGIBMGA002640 (annotated as 26S protease regulatory subunit 7-like isoform X1 and ubiquitin carboxyl-terminal hydrolase-like isoform X1, respectively) was up-regulated in the *cot* mutant after high-temperature treatment. BGIBMGA002640 encodes a deubiquitinating enzyme. The 26S protease regulatory subunit is reportedly a part of the 26S proteasome and the deubiquitinating enzymes have important functions in the ubiquitin–26S proteasome system (UPS)^41^,^42^. The two proteins were up-regulated in the *cot* mutant when exposed high temperature, suggesting that they play important roles in the degradation of the mutated Bmsei protein by the UPS.

*Drosophila DJ-*1β mutants show a significant loss of locomotor ability and *DJ-*1β plays a role in protecting the organism against oxidative stress^43^, indicating that *DJ-*1β has a leading role in behavior. DJ-1β was up-regulated when the *cot* mutants were exposed to high temperature, and might therefore be associated with the epilepsy phenotype.

When treated with high temperature, the *cot* mutant manifests an epilepsy phenotype, including strong contractions, rolling, vomiting, and a temporary lack of movement. Because this closely resembles the symptoms of human epilepsy reported in earlier studies, we speculated that the mutated gene in *cot* is homologous to the genes affected in human epilepsy. Our present results show that the mutated gene in the *cot* mutant (*Bmsei*) is highly homologous to hERG. Inherited mutations in *hERG* cause long QT syndrome 2 (LQT2), a disorder associated with cardiac arrhythmia^44^. The seizure phenotype of LQT2 is caused by abnormal concentrations of K^+^ in the nervous system, suggesting that *hERG* mutations predispose patients to epilepsy and the lethal cardiac arrhythmia of LQT2^45^,^46^. In 2012, Zamorano-Leon found that the *hERG* gene is a potential link between epilepsy and LQT2 syndrome^46^. In our previous study, we showed that the *cot* mutant not only manifests the epilepsy phenotype, but importantly, also displays an increased heartbeat when exposed to high temperature^30^. An LC–MS–MS analysis of neonatal larvae demonstrated that the level of BGIBMGA001031 (troponin I-like isoform X3) was also significantly up-regulated in the *cot* mutant. TnI is a myofibrillar protein that regulates the interaction of actin and myosin, and the levels of TnI are elevated in patients with seizures^47^. TnI is also used as a highly specific and sensitive serum biomarker for the diagnosis of acute myocardial infarction^48^. Therefore, the *cot* mutant might provide a model of LQT2 for drug screening, although electrocardiography has not been performed in the *cot* mutant. This work will be undertaken in our future research. We hope that the *cot* mutant will become a model for studying the relationship between seizure and cardiac arrhythmia, and will contribute to future research into human disease.

In conclusion, mutated Bmsei is responsible for the *cot* mutant, which displays an epilepsy phenotype that closely resembles human epilepsy after treatment with high temperature. This extensive protein profile provides insights that extend our understanding of the molecular mechanisms underlying the behavior of the silkworm. Our data suggest that the *cot* mutant is an invaluable tool for understanding the relationship between behavior and high-temperature stimulation in insects, and is also a potential model for the future study of human epileptic diseases.

## Methods

### Silkworm Strains

The WT silkworm strain (Dazao), the transparent cuticle mutant *ok*, the contractile mutant strain *cot*, and other *B. mori* strains were kindly provided by the Silkworm Gene Bank of Southwest University, China. The *cot* strain is homozygous and the heterozygous *cot*/+ strain was generated by mating +/+ (Dazao) and the *cot*/*cot* genotype. The silkworm larvae were reared on mulberry leaves. A female *cot* moth was mated with a male Dazao moth to obtain F_1_ progeny (*cot*/*cot* ♀ × +/+ ♂). For positional cloning, we used single-pair backcrossed (BC_1_) individuals from the *cot/cot* ♀ × F_1_♂ cross. Individuals with genotypes *ok/ok*, *cot/cot* or *ok/ok*, *+/+* were obtained with a previously described method^30^, and used for subsequent experiments.

### Behavioral Observations

Our previous studies showed that *cot* is a temperature-sensitive mutant with a seizure phenotype^30^, so it was treated at different temperatures. To separate the *cot*/+ and *cot/cot* genotypes, larvae of the different genotypes were exposed to 25 °C, 30 °C, 35 °C, 40 °C, 45 °C, or 48 °C in an electrothermal incubator and maintained for 5 min at each temperature. Their behavior was recorded with a camera (Canon xi810).

### Isolation and Characterization of the *cot* Mutant

On day 5 of the fifth instar, BC_1_ silkworms with the *cot/cot* and *cot/+* genotypes were placed in an incubator at 42 °C. Strong contraction, rolling, and vomiting were observed within 5 min, identifying the *cot* homozygotes for use in positional cloning. The silkworms that did not show the *cot* phenotype after 10 min were deemed to be *cot/+*. The individuals were kept in single bags, placed in liquid nitrogen for a few minutes, and stored at –80 °C.

### Screening SNP Markers

Genomic DNA was isolated with an automated DNA isolation system (Kurobo-PI1200, Japan), according to the manufacturer’s instructions. Appropriate SNP markers in linkage group 15 were used to construct a low-density linkage map of *cot*^49^,^50^. The SNP markers were taken from the resequence map of 40 *B. mori* strains^51^. The PCR products were sequenced using the BigDye Terminator v3.1 Cycle Sequencing Kit (Applied Biosystems) and a DNA analyzer (model 3730; Applied Biosystems). The software Sequencer 4.0 was used to screen the SNP markers between parents (Dazao and *cot* parents) and F_1_. The primers for the SNP markers are given in Supplementary information, Table S4.

### Annotating Candidate Genes within the *cot* Linkage Region

We searched the *cot*-linked region in the SilkDB (http://silkworm.swu.edu.cn/silkdb) and KAIKO base (http://sgp.dna.affrc.go.jp/KAIKObase/) and downloaded the sequences of candidate genes. The domains were predicted online using SMART (http://smart.embl-heidelberg.de) and SOSUI (http://harrier.nagahama-i-bio.ac.jp/sosui/sosui_submit.html). We obtained the amino acid sequences of homologous candidate genes from the National Center for Biotechnology Information database (http://www.ncbi.nlm.nih.gov/Entrez/).

### RT-PCR and qRT–PCR

The fifth instar larvae (5 day) were dissected on ice, and the main tissues such as head, epidermis, fat body, midgut, anterior silk gland, middle silk gland, posterior silk gland, sexual gland and ganglion were isolated. For expression profile of *Bmsei* in the embryo, samples of WT on each day were collected during whole embryo stage. Neonate larvae and the heads of different developmental stages were also collected for expression profile of *Bmsei* in three genotypes larvae (*cot*/*cot*, *cot*/+ and +/+) at different stages. Total RNA was extracted using TRIzol (Invitrogen) from the indicated stages and tissues of the different *cot* genotypes and WT. cDNA libraries were constructed according to the instructions of the PrimeScript^TM^ RT reagent Kit (RR037A, Takara). The PCR cycling conditions were 94 °C for 5 min, followed by 25 (or 35) cycles of 94 °C for 10 s, 60 °C for 15 s, and 72 °C for 1 min, followed by extension for 72 °C for 7 min. The ribosomal protein L3 (*BmRpL3*) was used as the internal control for normalization. The PCR products were sequenced with an Applied Biosystems DNA analyzer (ABI 3730lx). All qRT–PCR was performed with a previously described method^52^. The primers are listed in Supplementary information, Table S5.

### Identifying the *cot* Mutation Site

The primers were designed according to genomic sequences and *Bmsei* mRNA, and are listed in the Supplementary information, Table S5. The total RNA was extracted with TRIzol Reagent (Invitrogen) from the heads of each strain with genotype *cot/cot, cot/*+ and +/+ on the day 5 of fifth instar and reverse transcribed with M-MLV Reverse Transcriptase (Promega). The PCR cycling conditions were the same as described above.

### Immunofluorescence

Two Bmsei peptides (N → C: CLRALSLRFKTTHAPP and LGKDDIFGENPC) were synthesized and used to prepare a monoclonal antibody directed against Bmsei. Larval ganglia were dissected on ice on day 5 of the fifth instar. For the whole-ganglion immunofluorescence analysis, the larval ganglia were fixed immediately in 4% paraformaldehyde containing 3.7% PIPES, 0.5 mol/L EGTA, 1 mol/L MgSO4, at pH 7.0 (adjusted with 1 M KOH). Whole ganglia were permeabilized for 60 min at room temperature in PBS containing 0.5% NP-40, blocked in PBS containing 10% Triton X-100 and 0.2% bovine serum albumin at room temperature for 2 h, and incubated with rabbit anti-Bmsei antibody (1:100) in 10 mM PBS overnight at 4 °C. The whole ganglia were washed with PBS and incubated with fluorescein -isothiocyanate-labeled goat anti-rabbit IgG (H+L) antibody (1:500; Beyotime) in 10 mM PBS for 2 h at room temperature. To analyze the immunofluorescence in ganglion sections, the ganglia were fixed for 2 h with freshly prepared 4% (w/v) paraformaldehyde in PBS at 4 °C. After dehydration by passage through an ethanol gradient, the tissues were embedded in paraffin, processed into 5 μm serial sections, and treated at 60 °C for 1 h. The sections were incubated for 30 min in 10 mM citrate buffer (pH 6.0) at 95 °C for antigen retrieval. The samples were blocked with 10% (w/v) bovine serum in 10 mM PBS (pH 7.5) at 37 °C for 30 min. Anti-Bmsei and secondary antibodies were used as described above. To localize the Bmsei protein, the sections were stained with 4′,6-diamidino-2-phenylindole for 10 min. The fluorescent images were acquired with a confocal microscope (FV1000; Olympus).

### Immunoblotting

After hatching, whole larvae were homogenized in PBS (0.8% NaCl, 0.02% KCl, 0.144% Na_2_HPO4, 0.024% KH_2_PO4, 20 mM protease inhibitor cocktail, and 20 mM PMSF, pH 7.5). The protein concentrations were measured with the Bradford method^53^ and 150 μg of protein was separated with 6% SDS-PAGE and transferred onto PVDF membranes (Roche). The membranes were blocked with 5% nonfat dry milk overnight at 4 °C, before incubation with rabbit anti-Bmsei antibody (1:10,000) for 2 h at 37 °C, and were then incubated with goat anti-rabbit horseradish-peroxidase-conjugated secondary antibody (1:20000, A6154; Sigma) for 1 h at 37 °C. To normalize Bmsei expression to a reference protein, the membranes were also probed with a mouse anti-α-tubulin antibody (1:20,000, AT819; Beyotime). A secondary peroxidase-conjugated anti-mouse IgG antibody produced in goats (A2554, Sigma) was used at 1:20,000. Final visualization was achieved with the Super West Femto Chemiluminescent Substrate (30945; Thermo Scientific). The blots were analyzed and quantified with the Quantity One 4.6.2 software (Bio-Rad).

### RNAi Experiments

To synthesize *Bmsei* dsRNA, a template DNA fragment was prepared as previously described^54^. A 387-bp fragment of *Bmsei* was amplified from the head cDNA of a one day 5, fifth instar larva. The PCR products were cloned into the pGEM-T easy vector (Promega) and confirmed with sequencing. The plasmids were linearized with *Pst*I. The products were purified with absolute alcohol and used for dsRNA synthesis using the T7 RiboMAX™ Express RNAi System (Promega), according to the manufacturer’s instructions. *EGFP* dsRNA was synthesized as the control, as described previously^55^. The dsRNA was injected into WT eggs within 8 h of oviposition using a microinjector (IM300; Eppendorf). The eggs were incubated at 25 °C until hatching. The neonatal larvae were exposed to 42 °C for 5 min and recorded with a video camera.

### Statistical analysis

Using GraphPad Prism 5, data were analyzed with an analysis of variance (ANOVA) with Tukey’s multiple comparison test for multiple comparisons. Probability values of less than 0.05 were considered significant and tests were performed two-sided. Data are presented as mean and error bars depict the standard error of the mean (s.e.m.).

### Injection of Potassium Channel Blockers and Gentamicin

The potassium channel blockers tetraethylammonium chloride (TEA) and BaCl_2_ were injected at various concentrations into WT larvae and moths. The larvae and moths were approximately the same weight and were injected in the stoma or intersegmental membrane using a glass needle with 5 μL of solution containing different concentrations of blockers. Similarly, different concentrations of gentamicin were injected into the contractile mutants. All the phenotypes were recorded with a camera (Canon xi810).

### LC–MS–MS and Data Analysis

The proteins were extracted from neonatal larvae using the method described above in “Immunoblotting”. According to a previously described method^56^,^57^, 150 μg of protein in PBS was digested and subjected to Thermo Scientific Q Exactive mass spectrometer operating in data-dependent mode. The raw data were then analyzed with MaxQuant version 1.3.0.1^58^. The peptide and protein data are given as Supplementary information, Tables S6 and S7. To compare the protein abundances across different samples, label-free quantification was used to determine the protein intensities^59^. To ensure the reliability of the data, the proteins with an intensity >0 in all samples were selected for subsequent analysis. The relative intensities in the high-temperature-treated group and the normal group were compared for each strain. The expression intensity ratios (high temperature/room temperature) that were >2.0 or <0.5 were set as the thresholds indicating significant change. Proteins for which this ratio was >2.0 in the *cot* strain and <2 in the control strains (*ok* and Dazao) were regarded as upregulated, whereas those with ratios <0.5 in the *cot* strain and >0.5 in the control strains were regarded as downregulated. The proteins of prominent changes were aligned with BLASTp and the results were analyzed for mapping, annotation, enzyme code, and combined graphing using the BLAST2GO software (version 3.0, http://www.blast2go.com), with the default settings.

## Additional Information

**How to cite this article**: Nie, H. *et al.* Functional Loss of *Bmsei* Causes Thermosensitive Epilepsy in Contractile Mutant Silkworm, *Bombyx mori*. *Sci. Rep.* **5**, 12308; doi: 10.1038/srep12308 (2015).

## Supplementary Material

Supplementary Video S1

Supplementary Information

Supplementary Table S2

Supplementary Table S3

Supplementary Table S6

Supplementary Table S7
