# Supplementary material for: Functional Loss of Bmsei Causes Thermosensitive Epilepsy in Contractile Mutant Silkworm, Bombyx mori
Source: Sci Rep. 2015 Jul 22;5:12308. doi: 10.1038/srep12308 (PMC4510522; doi:10.1038/srep12308)
Supplement: Supplementary Information [file srep12308-s2.pdf]

Functional Loss of *Bmsei* Causes Thermosensitive Epilepsy in Contractile Mutant Silkworm, *Bombyx mori*

Hong-Yi Nie[1,2,3,4], Ting-Cai Cheng[1,2,3], Xiao-Feng Huang[1], Meng-Ting Zhou[1], Yin-Xia Zhang[1], Fang-Yin Dai[1,2,3], Kazuei Mita[1], Qing-You Xia[1,2,3*], Chun Liu[1,2,3*]

[1]State Key Laboratory of Silkworm Genome Biology, Chongqing 400716, China

[2]the Key Sericultural Laboratory of the Ministry of Agriculture, Southwest University, Chongqing 400716, China

[3]College of Bio-Technology, Southwest University, Chongqing 400716, China

[4]College of Bee Science, Fujian Agriculture and Forestry University, Fujian Fuzhou 350002, China

* To whom correspondence should be addressed: Chun Liu, State Key Laboratory of Silkworm Genome Biology, Southwest University, Chongqing, 400716, China, Tel.:+86-23-68251753, E-mail：mlliuchun@163.com. Qing-You Xia, State Key Laboratory of Silkworm Genome Biology, Southwest University, Chongqing, China, Tel:+86-23-6825-1996; Fax: 86-23-6825-1228; E-mail: xiaqy@swu.edu.cn

Supplementary information, Figure S1

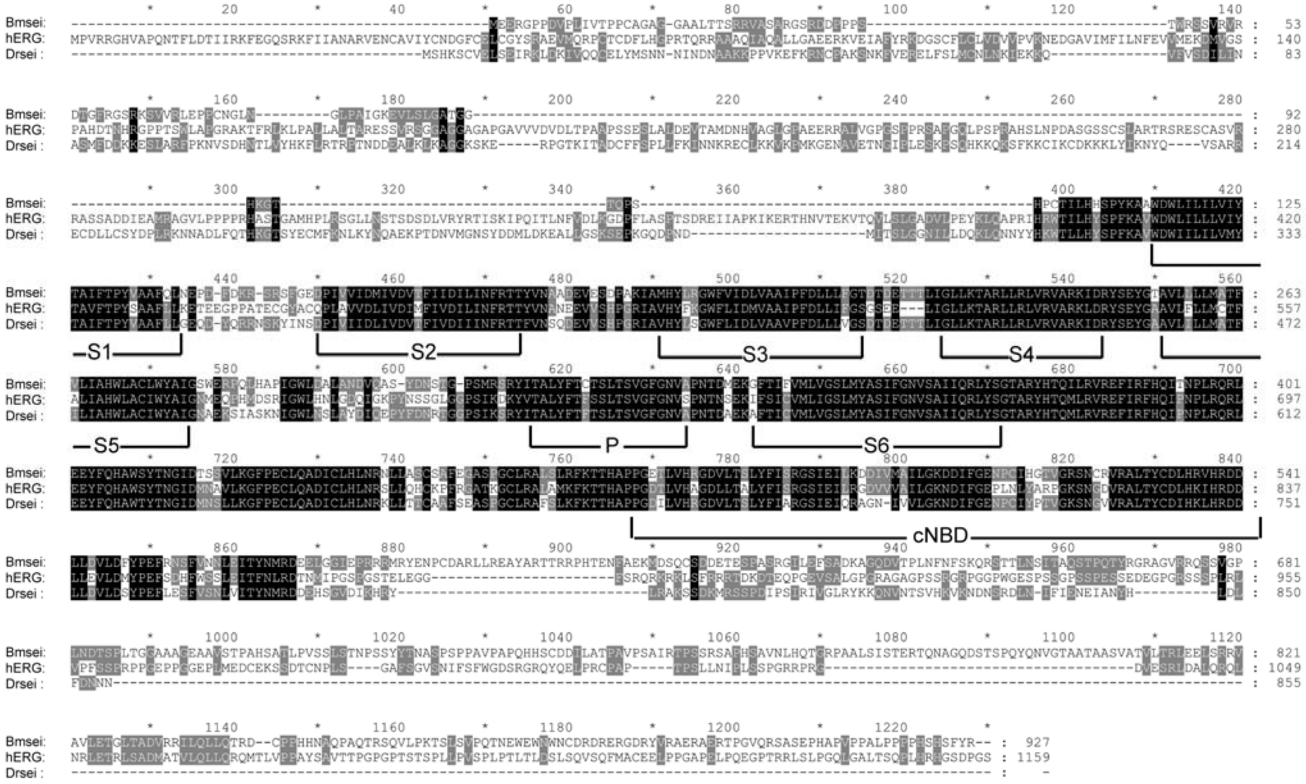

Supplementary Figure 1 Amino acid sequence alignment of deduced Bmsei sequence with Drsei and hERG. Bmsei amino acid sequence compared with the HERG potassium channel subunit and seizure of *Drosophila* (Drsei). Underlined: six hydrophobic domains (S1–S6), pore (P), and cyclic nucleotide-binding domain (cNBD). Black, conserved residues; gray, residues common between two sequences.

Supplementary information, Figure S2

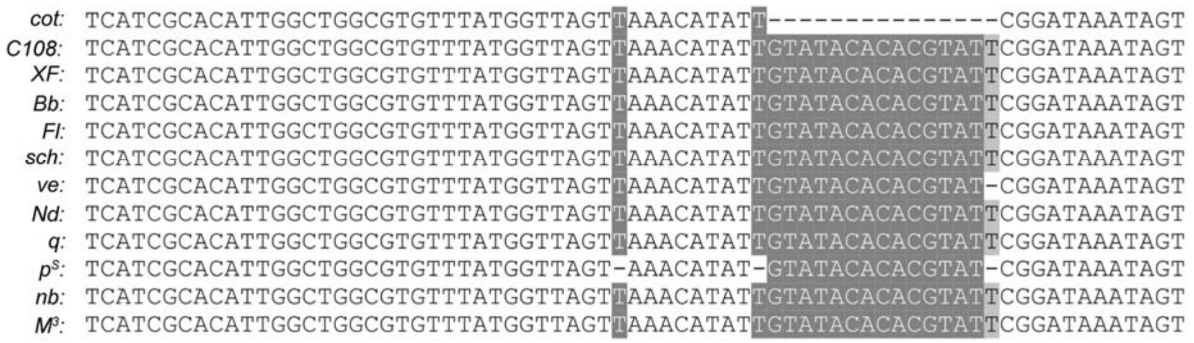

Supplementary Figure 2 Multiple sequence alignment of the fifth intron of *Bmsei* from 12 silkworm strains. Region harboring the 15 bp sequence in the fifth intron of *Bmsei* amplified by PCR using 12 genomic DNAs including from *cot* mutant. PCR products were sequenced. The 15 bp deletion was unique to *cot* mutants. Strains: contractile (*cot*), *C108*, Xiafang (*XF*), Bibo (*Bb*), Flossy (*Fl*), sex-linked chocolate (*sch*), varnished eye (*ve*), quail (*q*), Striped (p^S), narrow breast (*nb*), M^3.

Supplementary information, Figure S3

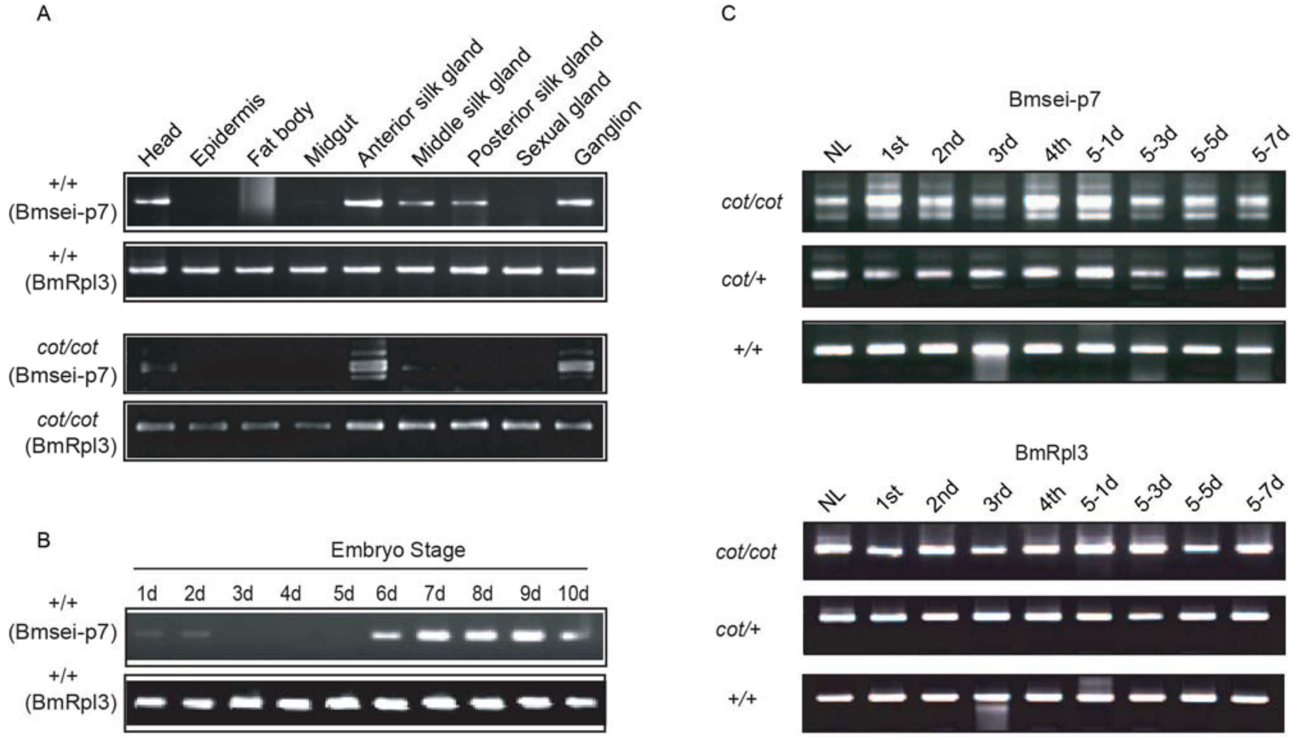

Supplementary Figure 3 Expression patterns of *Bmsei* by RT-PCR. (A) Spatial expression of *Bmsei* on day 5 of the fifth instar larval stage in +/+ and *cot/cot*. (B) Expression profile of *Bmsei* in the embryo stage in wild type. (C) Expression profile of *Bmsei* in larvae at different stages and three genotypes, *cot/cot, cot/+* and +/+ (Dazao). Developmental stages: NL, neonate larvae; 1 st, day 1 of first instar; 2 nd, day 1 of second instar; 3 rd, day 1 of third instar; 4 th, day 1 of fourth instar; 5-1 d, day 1 of fifth instar; 5-3 d, day 3 of fifth instar; 5-5 d, day 5 of fifth instar; 5-7 d, day 7 of fifth instar.

Supplementary information, Figure S4

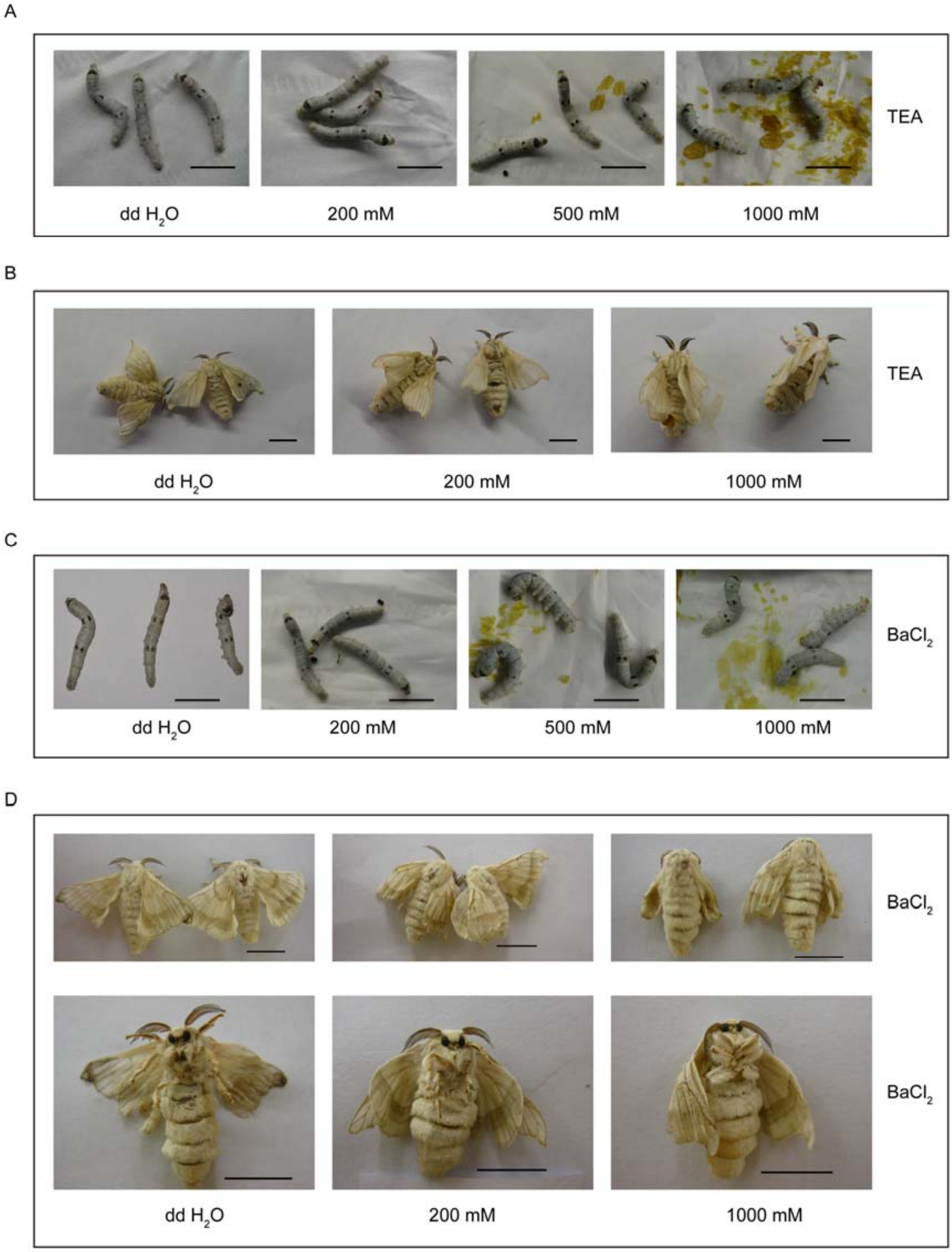

Supplementary Figure 4 Phenotype of WT with TEA and $BaCl_2$ treatment of larvae and moths. (A and C) WT larvae were injected with 0 mM, 200 mM, 500 mM, or 1000 mM TEA (upper) or $BaCl_2$ (lower). (B and D) Moths were injected with 0 mM, 200 mM, 1000 mM TEA (upper) or $BaCl_2$ (lower). Scale bar, 1 cm.

Supplementary information, Figure S5

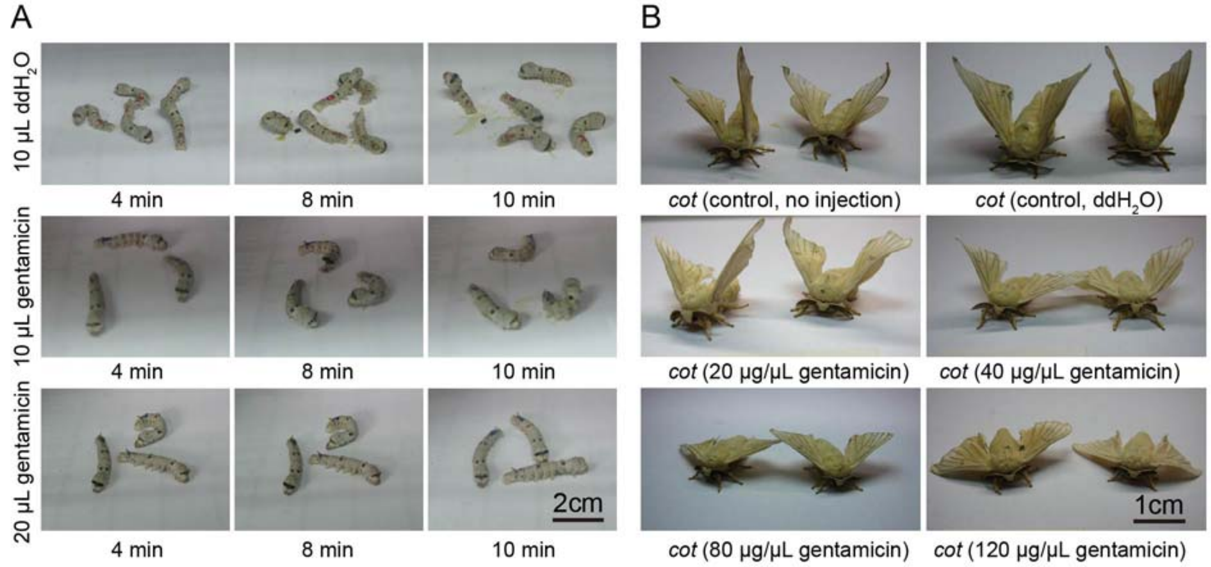

Supplementary Figure 5 Gentamicin partly rescues the *cot* phenotype. (A) Phenotype of larva (day 2 of fifth instar) injected with different gentamicin dose when treated with high temperature at 35 ºC. Larva were injected 10 µL ddH_2O (upper panel), 10 µL gentamicin (40 µg/µL, middle panel) and 20 µL gentamicin (40 µg/µL, lower panel,) on day 2 of fifth instar. Then the phenotypes were observed at 4 min, 8 min and 10 min after treated with high temperature at 35 ºC. (B) Gentamicin at different concentrations (20 µg/µL, 40 µg/µL, 80 µg/µL and 120 µg/µL) was injected into contractile mutants and treated at 42ºC.

Supplementary information, Table S1 List of ten candidate genes' domain and function

Gene	Number of animo acid	Domain	Function
BGIBMGA007797	566	Zinc finger	Contain multiple finger-like protrusions and make tandem contacts with their target molecule
BGIBMGA007730	1357	Protein kinase domain	Phosphorylation, embryonic development, physiological responses and in the nervous and immune system;
		CNH domain	Macromolecular interactions
BGIBMGA007796	132	Prefoldin_2	Interacts exclusively with type II chaperonins, hetero-oligomers
BGIBMGA007731	304	No	/
BGIBMGA007795	113	Ribosomal protein L7Ae/L30e/S12e/Gadd45 family	Catalyse mRNA-directed protein synthesis in all organisms
BGIBMGA007732	232	Protein tyrosine phosphatase	Remove phosphate groups from phosphorylated tyrosine residues on proteins
BGIBMGA007733	486	No	/
BGIBMGA007734	428	Bystin	Implantation and trophoblast invasion
BGIBMGA007794	898	Transmembrane ion channel family;	The family of tetrameric sodium, potassium and calcium ion channels;
		Cyclic nucleotide-binding domain	Binding cyclic nucleotides (cAMP or cGMP)
BGIBMGA007735	1167	Transmembrane domain of ABC transporters;	Transmembrane structural unit of ATP-binding cassette transporter;
		ATP-binding domain of ABC transporters	Exporting or importing of a wide variety of substrates

Note: Number of animo acid, Domain and Function for each gene is shown based on the silkDB (http://silkworm.swu.edu.cn/silkdb/) and Pfam blast on line. “NO” indicates that the gene didn’t contain any domain, and the virgule indicates no report of the function.

Supplementary information, Table S4 Primers for SNP markers used for linkage analysis of *cot*

NO.	Name of SNP marker	Forward primer	Reverse primer
1	15035_3.7	TCGTTCCTTGAGAGTGTCCA	TGCACCTTCAGACTTGTACGTT
2	15105_9.5	TAATGAAGCGTGGAGATGGG	CGTTAATGCGTTGTAATGCG
3	15006_16.4	TTTTGAGGGGAGAAACACAA	ACGGTTGCCCTACAAGGTTA
4	15034_16.4	AATACCCGCGTGCTCACTAC	TTCTTTTATGTTTGGCAAATCG
5	15107_17.4	CCCCGGACAATACAGTCCTA	GGAATTGTCCCACACTCCAC
6	15100_27.2	ACAGGACCACCGAGTCAGAT	AGACCCGTGTCCAGACAGAT
7	15007_36.0	TTTCACGATCTTAATCGTCTTG	GTTCGCTCGTACTTTGGCTA
8	15057_36.6	AGCATAATTTGATTTGAGTGATGTG	TAGTGTGCCTGGTTGTGGTT
9	15011_19.0	ACGTCGATCATGACTTTCCC	ATCGCGAATTGCTAATGCTT
10	15003_19.5	CCGAGTAGAGTTCCATTGCG	CGACTGGTCCCATTGTTTCT
11	15078_21.5	GATCCTGGCTCAGGACGG	GGTACTTTGTTGTTGGTGGCA
12	15002_22.0	TGACTCTCTTTGCGTAATTTCTTT	TTTCGATGAAATGAGGGGAC
13	15021_25.1	TCAAGACGCGAACATTGGTA	TGGAATGGCAACGATGAGTA
14	15028_25.6	AAGGTCAAATTAACTACCTACTTAGCA	TCCGAGAATGTGACATGTGATT
15	chr15_Bm_scaf3_846621	CGTTCAAATACCTTGGGAGA	GCCCTTCGTTGAGTGAGTT
16	chr15_Bm_scaf3_1251147	TGTTATTTCCGTTTCACCAG	TGTCCAGGCATAAAGCACC
17	chr15_Bm_scaf3_1684999	CGATAGAAAAGCAAATGACG	AGTCGTGCTTATCACATTCG
18	chr15_Bm_scaf3_1743581	CTGCGTATCACGGTTCCTT	TCACCCTGAACACCTTACCT
19	chr15_Bm_scaf3_1002769	TTCCCTTGAATGATAAGTTGC	CCATGTGTTGACAGTTTGAAGAA
20	chr15_Bm_scaf3_1114648	CCTTTTGACTTTTCAGTTGGA	CCACATTCACATTTTCTCCC
21	chr15_Bm_scaf3_1398039	CACCGCTGTTGTTAGATAGG	TTCGTATCCTTGTATCCGTCC
22	chr15_Bm_scaf3_1504561	CCTCTGACAATCATTTGGACT	TGATAAGGATAGCACGAAACAG
23	chr15_Bm_scaf3_1586251	CGACGACAACTCGCTCGAACT	CCGATTGACTTGAAACTTGGT
24	chr15_Bm_scaf3_1101731	ATTCATTTCCCCTTCTATCCA	GGCAGTAGTGTTCGATAATGTG
25	chr15_Bm_scaf3_1271214	TTCAAGTCGCTATTGGAGTAA	TGTTATTTGTTGCTTTCCAGTC

Supplementary information, Table S5 Primers for genomic PCR, RT-PCR and qRT-PCR

No.	Primer name	Forward primer	Reverse primer
1	*Bmsei* -p1	GTCGGAGCACGGTGGGTT	CTGCGGTCGCTCCCAACT
2	*Bmsei* -p2	ACCGCTGTCCTTCTATTGCTC	GCGAAATAAAGTATAGGGAGGTCA
3	*Bmsei*-p3	TCCCCGAGTGCCTCCAAG	CGCAGGAATGATGTTGTGGC
4	*Bmsei*-p4	CAGCGCCACAACATCATTCC	GCCCGCCTCACACCTACC
5	*Bmsei*-p5	GGTGGGTTTCGGTGAGGAC	GATGGCTGGTAGTCCGTTG
6	*Bmsei*-p6	GGTGGGTTTCGGTGAGGAC	CGGATTCCACTTCGTCAGC
7	*Bmsei*-p7	GGCGAAGATCCCATTGTAG	AACCTTTTTCCATGTCTGTGT
8	*Bmsei*-p8	ACCGCTGTCCTTCTATTGC	TTCTCGCCGAAAATGTCA
9	*Bmsei*-p9	ATTCAAGACAACTCACGCTCCA	TTTGTCCGCAGAAAACTCG
10	*Bmsei*-p10	ATGCGCTACGAGAATCCTTG	GCCTGCTTAGTTCTTCCAATCT
11	*Bmsei*-p11	CGCCATCTTCTCGTTCTGC	CCTGTAGAACGAGTGCGAGT
12	*Bmsei*G-1	AGTGTAAGCGGAAGTAATAGTCG	CTTATGCGTTGCTTGCTTC
13	*Bmsei*G-2	TCAGAAAATTCGACTGGCTAT	ATAAGGGCTCACTTCATCG
14	*Bmsei*G-3	ATTTTGCTTAGTTAGCGTGAT	TTCGACTTCGCGTTACATAAT
15	*Bmsei*G-4	TATTCATGTTTTCAGAGCCCTC	ATTTTTGGACCCTTTTTCACA
16	*Bmsei*G-5	TAAGTGAACTCTTTGTCCAGG	TGTGATGCATTTCATTCTTAAC
17	*Bmsei*G-6	AGTGCATGATATTATGACGTTGT	AGGAAAGTTCTGGCGTCGTT
18	*Bmsei*G-7	CTGGGACATTTAGACGGACA	ATAACTATTTATCCGAATACGTGTGTA
19	*BmRpl3*-1	TCGTCATCGTGGTAAGGTCAA	TTTGTATCCTTTGCCCTTGGT
20	*BmRpl3*-2	CGGTGTTGTTGGATACATTGAG	GCTCATCCTGCCATTTCTTACT
21	*Bmsei*-q8	ATCTTCGTGGCTGGTTCGTCATA	CCAACTCCCGATAGCATACGTAG
22	*Bmsei*-q9	ATCTTCGTGGCTGGTTCGTCATA	ACTCCCGATAGCATACCATAAAC
